# Multicenter phase 1/2 study of forodesine in patients with relapsed peripheral T cell lymphoma

**DOI:** 10.1007/s00277-018-3418-2

**Published:** 2018-07-05

**Authors:** Dai Maruyama, Kunihiro Tsukasaki, Toshiki Uchida, Yoshinobu Maeda, Hirohiko Shibayama, Hirokazu Nagai, Mitsutoshi Kurosawa, Yoko Suehiro, Kiyohiko Hatake, Kiyoshi Ando, Isao Yoshida, Michihiro Hidaka, Tohru Murayama, Yoko Okitsu, Norifumi Tsukamoto, Masafumi Taniwaki, Junji Suzumiya, Kazuo Tamura, Takahiro Yamauchi, Ryuzo Ueda, Kensei Tobinai

**Affiliations:** 1 Department of Hematology, National Cancer Center Hospital, 5-1-1 Tsukiji, Chuo-ku, Tokyo, 104-0045 Japan; 2 Department of Hematology, National Cancer Center Hospital East, Kashiwa, Japan; 3 Department of Hematology and Oncology, Japanese Red Cross Nagoya Daini Hospital, Nagoya, Japan; 4 Okayama University Hospital, Okayama, Japan; 5 Osaka University Hospital, Osaka, Japan; 6 Clinical Research Center, National Hospital Organization Nagoya Medical Center, Nagoya, Japan; 7 Hokkaido Cancer Center, Sapporo, Japan; 8 Kyushu Cancer Center, Fukuoka, Japan; 9 The Cancer Institute Hospital, Tokyo, Japan; 10 Tokai University Hospital, Isehara, Japan; 11 Shikoku Cancer Center, Matsuyama, Japan; 12 Kumamoto Medical Center, Kumamoto, Japan; 13 Hyogo Cancer Center, Akashi, Japan; 14 Tohoku University Hospital, Sendai, Japan; 15 Gunma University Hospital, Maebashi, Japan; 16 University Hospital, Kyoto Prefectural University of Medicine, Kyoto, Japan; 17 Shimane University Hospital, Izumo, Japan; 18 Fukuoka University Hospital, Fukuoka, Japan; 19 Fukui University Hospital, Fukui, Japan; 20 Aichi Medical University, Nagakute, Japan

**Keywords:** Forodesine, Peripheral T cell lymphoma, Overall response rate, Progression-free survival, Purine nucleoside phosphorylase inhibitor

## Abstract

Peripheral T cell lymphomas are an aggressive group of non-Hodgkin lymphomas with poor outcomes for most subtypes and no accepted standard of care for relapsed patients. This study evaluated the efficacy and safety of forodesine, a novel purine nucleoside phosphorylase inhibitor, in patients with relapsed peripheral T cell lymphomas. Patients with histologically confirmed disease, progression after ≥ 1 prior treatment, and an objective response to last treatment received oral forodesine 300 mg twice-daily. The primary endpoint was objective response rate (ORR). Secondary endpoints included duration of response, progression-free survival (PFS), overall survival (OS), and safety. Forty-eight patients (median age, 69.5 years; median of 2 prior treatments) received forodesine. In phase 1 (*n* = 3 evaluable), no dose-limiting toxicity was observed during the first 28 days of forodesine treatment. In phase 2 (*n* = 41 evaluable), the ORR for the primary and final analyses was 22% (90% CI 12–35%) and 25% (90% CI 14–38%), respectively, including four complete responses (10%). Median PFS and OS were 1.9 and 15.6 months, respectively. The most common grade 3/4 adverse events were lymphopenia (96%), leukopenia (42%), and neutropenia (35%). Dose reduction and discontinuation due to adverse events were uncommon. Secondary B cell lymphoma developed in five patients, of whom four were positive for Epstein-Barr virus. In conclusion, forodesine has single-agent activity within the range of approved therapies in relapsed peripheral T cell lymphomas, with a manageable safety profile, and may represent a viable treatment option for this difficult-to-treat population.

## Introduction

Peripheral T cell lymphomas (PTCLs) are an aggressive group of non-Hodgkin lymphomas (NHLs), accounting for 5–10% of all NHLs in Western countries and approximately 20% of NHLs in Japan [1, 2]. The most common PTCL subtypes are PTCL, not otherwise specified (PTCL-NOS); anaplastic large cell lymphoma (ALCL); and angioimmunoblastic T cell lymphoma (AITL) [3, 4]. Newly diagnosed PTCL is most frequently treated with cyclophosphamide, doxorubicin, vincristine, and prednisone (CHOP) or CHOP-like chemotherapies [5–8]. Although most patients respond initially, outcomes remain poor except in patients with anaplastic lymphoma kinase (ALK)-positive ALCL [5]. Median survival in a cohort of 153 North American patients with first-relapsed/refractory PTCLs was 5.5 months, underscoring the need for new treatments in this setting [9].

Prior to forodesine, several agents were approved for treatment of relapsed/refractory PTCL, including the folate antagonist pralatrexate [10] and the histone deacetylase inhibitors romidepsin [11] and belinostat [12] in the USA, and chidamide in China [13]. Additionally, the anti-CC chemokine receptor 4 monoclonal antibody mogamulizumab was approved in Japan for treatment of anti-CC chemokine receptor 4-positive relapsed/refractory PTCL [14], and the anti-CD30 antibody-drug conjugate brentuximab vedotin was approved for treatment of relapsed/refractory ALCL [15, 16]. Objective response rates (ORRs) for these agents in relapsed/refractory PTCL generally ranged from 25 to 35% [10–14], with brentuximab vedotin producing an ORR of 86% in ALCL [15] but 21% in non-ALCL PTCL [16].

Children born deficient in purine nucleoside phosphorylase (PNP) have reduced T cell counts, suggesting that PNP may be a target for treatment of T cell-mediated diseases [17]. Forodesine (BCX1777, Immucillin-H) is a novel PNP inhibitor, 100 to 1000 times more potent than other agents of this class [18, 19]. By inhibiting PNP, forodesine augments 2′-deoxyguanosine (dGuo) levels in plasma and T cells. The enzyme 2′-deoxycytidine kinase, which is highly upregulated in malignant T cells, phosphorylates dGuo to form deoxyguanosine monophosphate and then 2′-deoxyguanosine triphosphate. Accumulation of 2′-deoxyguanosine triphosphate causes an imbalance of the deoxyribonucleotide pool leading to T cell apoptosis. Forodesine was tolerated at doses of 200–300 mg once daily and exhibited preliminary evidence of anti-tumor efficacy in patients with PTCLs or cutaneous TCLs [19, 20]. Pharmacokinetic/pharmacodynamic data in healthy volunteers (data on file) suggested that a regimen of 300 mg twice-daily would be tolerable and provide forodesine exposure necessary for improving efficacy. Therefore, we conducted a phase 1/2 study of forodesine 300 mg twice-daily in Japanese patients with relapsed PTCL.

## Patients and methods

### Study design

This multicenter, open-label study was conducted at 21 sites in Japan from January 2013 to February 2017. The study consisted of two parts: a phase 1 component designed to confirm the safety and tolerability of forodesine 300 mg twice-daily for 28 days in patients with relapsed/refractory PTCL and a phase 2 component designed to evaluate the efficacy and safety of this forodesine regimen in relapsed PTCL. The study was conducted in accordance with ethical principles of the Declaration of Helsinki and in compliance with International Council for Harmonization guidelines for Good Clinical Practice. The Institutional Review Boards of all participating institutions approved the study protocol, and all patients provided written informed consent. The study was registered at ClinicalTrials.gov (NCT01776411).

In phase 1, forodesine 300 mg (three 100-mg capsules) was given twice-daily after meals in a 28-day cycle. Phase 2 was initiated because none of the first three patients completing the 28-day cycle had dose-limiting toxicity (DLT; defined as treatment-related grade 3/4 non-hematologic toxicity excluding nausea, vomiting, or diarrhea or grade 4 neutropenia or thrombocytopenia lasting ≥ 7 days). During phase 2, patients received forodesine until disease progression (PD), unacceptable toxicity, or withdrawal of consent. Forodesine was stopped temporarily for ≤ 2 weeks in the event of DLT or if needed for management of adverse events (AEs). After recovery, a single-dose reduction to 200 mg twice-daily was allowed.

Patients had study visits on days 1 and 15 of cycles 1–4 and day 1 of subsequent cycles. After 22 patients completed 2 cycles, an interim efficacy analysis was conducted using a Simon minimax 2-step design to assess for futility (≤ 2 patients with objective responses); the study was to be terminated if futility was demonstrated. If futility was not demonstrated, the study was to be continued. Data cutoff was to be conducted when all the patients for efficacy evaluation completed the clinical study procedure by week 24. Based on data obtained until that time point, the primary analysis was to be conducted.

### Patients

Japanese patients aged ≥ 20 years with histopathologically confirmed PTCL were eligible if they had PD after ≥ 1 prior treatment and had achieved an objective response on their last treatment. PTCL was defined according to the 2008 WHO Classification [3] and included PTCL-NOS, AITL, ALCL (ALK-positive or ALK-negative), extranodal natural killer cell/TCL (nasal type), enteropathy-associated TCL, hepatosplenic TCL, subcutaneous panniculitis-like TCL, and transformed mycosis fungoides. The PTCL subtype was diagnosed in each institution from lesion biopsy specimens and confirmed by an Independent Pathology Review Committee. Eligible patients had an enlarged lymph node or extranodal mass that was measurable in two perpendicular directions by computed tomography, with the greatest diameter > 1.5 cm; Eastern Cooperative Oncology Group performance status 0 or 1; and adequate hematopoietic, liver, and kidney function.

Patients who had received chemotherapy, radiation therapy, or high-dose corticosteroids (prednisolone ≥ 10 mg/day or equivalent) ≤ 21 days before the first dose of study drug were excluded, as were patients with a history of central nervous system involvement, allogeneic hematopoietic stem cell transplantation, autologous hematopoietic stem cell transplantation ≤ 100 days before study drug, severe cardiovascular or pulmonary disease, uncontrolled diabetes, or positivity for hepatitis B virus surface antigen, anti-hepatitis C virus antibody, or anti-human immunodeficiency virus antibody. Pregnant and lactating women and patients of child-bearing potential unwilling to use adequate contraception were also excluded.

### Assessments

Tumor assessments were conducted after every 2 cycles for the first 24 weeks and then every 4 cycles. Efficacy was evaluated by an Independent Efficacy Assessment Committee (IEAC) according to revised International Working Group criteria [21], and classified as complete response (CR), partial response (PR), stable disease, or PD. The primary efficacy endpoint was IEAC-assessed ORR, consisting of the proportion of evaluable patients with CR or PR. Secondary efficacy endpoints included investigator-assessed ORR, duration of response (DoR), time to treatment failure, progression-free survival (PFS), and overall survival (OS). Primary analyses were conducted on data obtained by the time of data cutoff. In addition, final analyses on data from the entire period (including after the data cutoff) were also described for information purposes. For analyses other than the primary endpoint, results of final analyses were described.

### Safety assessment

Safety was evaluated throughout the study and was comprised of AE monitoring, laboratory testing, physical examinations, vital-sign measurements, and 12-lead electrocardiograms. Severity of AEs was graded using the National Cancer Institute Common Terminology Criteria for Adverse Events, version 4.0.

### Pharmacokinetics

The first seven patients comprised the full pharmacokinetics set; they received in-patient treatment for the first 4 days and had an additional visit on day 8. Blood samples were collected predose and at 1, 2, 4, 6, 8, and 12 h after the first dose on both days 1 and 15 and also predose on days 2, 3, 4, 8, 29, and 57. In all other patients, blood samples were collected predose for the first dose on days 1, 15, 29, and 57. Plasma forodesine concentrations were measured using a validated liquid chromatography-tandem mass spectrometry (LC/MS/MS) method, with a lower limit of quantitation of 2.5 ng/mL. Key pharmacokinetic parameters, including the observed maximum concentration (*C*_max_) and the area under the concentration-time curve to the last measurable drug concentration (AUC_last_), were determined by non-compartmental analysis using WinNonlin. Additional blood samples were collected at the above times for measurement of plasma dGuo concentrations by a validated LC/MS/MS with a lower limit of quantitation of 5.0 ng/mL.

### Statistical analysis

Efficacy was evaluated in the full analysis set consisting of patients who received ≥ 1 dose of forodesine and had a postbaseline efficacy assessment. Using the Simon minimax two-step design [22], a sample size of 40 evaluable patients in phase 2 would have 80% statistical power at a one-sided α of 0.05 for showing a threshold ORR of 10%, assuming an expected ORR of 25%. A one-sided binomial test was used to determine if the observed ORR was above the predefined 10% threshold rate. To account for potential non-evaluable patients, the target sample size in phase 2 was set at 43. Time-to-event parameters were evaluated using Kaplan-Meier methods [23]. Safety was assessed in all patients who received ≥ 1 dose of study drug. AEs were coded to the Medical Dictionary for Regulatory Activities–Japanese, version 18.1. Safety parameters were summarized using descriptive statistics.

## Results

### Patients

**Fig. 1:**
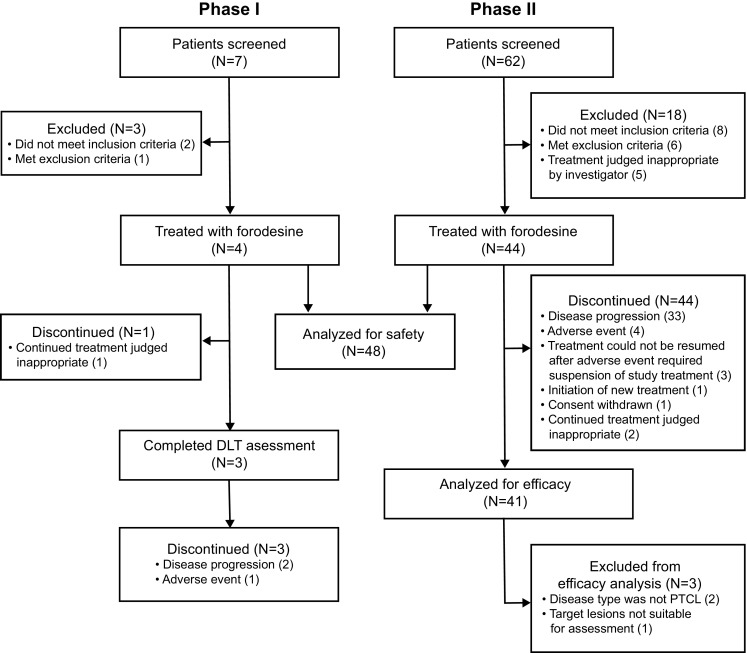
Disposition of patients in phase 1 and phase 2

Forty-eight patients received forodesine (4 in phase 1, 44 in phase 2) (Fig. 1). Overall, the most common reasons for discontinuation were PD (*n* = 35) and AEs (*n* = 8). The study cohort had a median age of 69.5 years (range, 32–79 years) and had received a median of two prior treatment regimens (range, 1–9); most had PTCL-NOS (46%) or AITL (40%) (Table 1).

**Table 1:** Demographics and baseline characteristics

Characteristic	Phase 1 (*n* = 4)	Phase 2 (*n* = 44)	Total (*n* = 48)
Age, years, median (range)	72.5 (42–76)	69.0 (32–79)	69.5 (32–79)
Sex, *n* (%)			
Male	1 (25)	30 (68)	31 (65)
Female	3 (75)	14 (32)	17 (35)
ECOG PS, *n* (%)			
0	2 (50)	27 (61)	29 (60)
1	2 (50)	17 (39)	19 (40)
Disease classification, *n* (%)			
Peripheral T cell lymphoma, NOS	1 (25)	21 (48)	22 (46)
Angioimmunoblastic T cell lymphoma	2 (50)	17 (39)	19 (40)
Anaplastic large cell lymphoma			
ALK-positive	1 (25)	0 (0)	1 (2)
ALK-negative	0 (0)	2 (5)	2 (4)
Extranodal T cell/NK cell lymphoma, nasal type	0 (0)	1 (2)	1 (2)
Transformed mycosis fungoides	0 (0)	1 (2)	1 (2)
Other^a^	0 (0)	2 (5)	2 (4)
Ann Arbor classification,^b^*n* (%)			
Stage I	0 (0)	1 (2)	1 (2)
Stage II	3 (75)	9 (20)	12 (25)
Stage III	0 (0)	20 (45)	20 (42)
Stage IV	1 (25)	13 (30)	14 (29)
LDH (baseline)			
Low/normal	2(50.0)	24 (55)	26 (54)
High	2(50.0)	20 (45)	22(46)
Prior treatment regimens			
Median (range)	2 (1–9)	2 (1–6)	2 (1–9)
Chemotherapy, *n* (%)	4 (100)	44 (100)	48 (100)
ASCT, *n* (%)	0 (0)	3 (7)	3 (6)
Radiation therapy, *n* (%)	1 (25)	5 (11)	6 (13)
Monoclonal antibody, *n* (%)	1 (25)	3 (7)	4 (8)
Corticosteroid, *n* (%)	1 (25)	2 (5)	3 (6)
Response to most recent treatment regimen, *n* (%)			
CR/CRu	3 (75)	20 (45)	23 (48)
PR	1 (25)	24 (55)	25 (52)

*ALK* anaplastic lymphoma kinase, *ASCT* autologous hematopoietic stem cell transplant, *CR* complete response, *CRu* unconfirmed complete response, *ECOG PS* Eastern Cooperative Oncology Group performance status, *LDH* lactate dehydrogenase, *NK cell* natural killer cell, *NOS* not otherwise specified, *PR* partial response, *SD* standard deviation

^a^Includes two cases judged to be plasmablastic lymphoma and follicular dendritic cell sarcoma, respectively, on the independent central pathology review

^b^Classification for PTCLs other than transformed mycosis fungiodes. The case of transformed mycosis fungiodes was stage IV by the ISCL-EORTC classification

Patients received forodesine for a median of 2.1 months (range, 0.2–36.0 months). Seventeen patients (35%) had a delay in forodesine dosing because of AEs, but only one patient (2%) had a dose reduction to 200 mg twice-daily (because of pneumonia). The mean daily dose of forodesine was 586.7 mg (standard deviation ± 37.2 mg).

### Safety

**Fig. 2:**
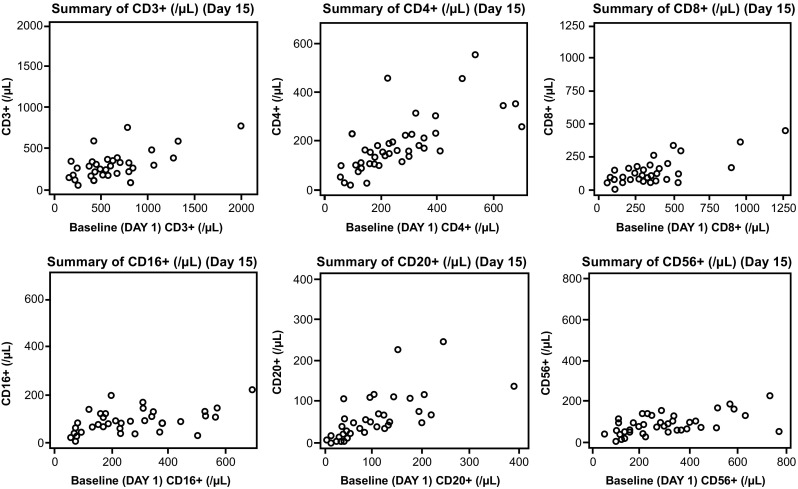
Scatterplots of lymphocyte subsets at baseline and on day 15

Lymphopenia occurred in all patients (grade 3/4 in 46 patients [96%]), with all evaluated lymphocyte subsets (CD3^+^, CD4^+^, CD8^+^, CD16^+^, CD20^+^, CD56^+^) showing reductions from baseline (Fig. 2). Other common grade 3/4 hematologic toxicities included leukopenia (42%), neutropenia (35%), and thrombocytopenia (25%; Table 2). Febrile neutropenia occurred in six patients (13%). Grade 3/4 non-hematologic toxicities were uncommon. Adverse events that resulted in discontinuation occurred in 11 patients (23%; only Epstein-Barr virus [EBV]-associated lymphoma (*n* = 2) led to discontinuation in > 1 patient). One patient in phase 2 died from disseminated intravascular coagulation and multiorgan failure, which was attributed to underlying disease and considered not related to forodesine.

**Table 2:** Adverse events regardless of causality occurring in ≥ 10% of patients

Adverse event	All grades, *n* (%)	Grade 3/4, *n* (%)
Hematologic toxicities		
Lymphopenia	48 (100)	46 (96)
Leukopenia	35 (73)	20 (42)
Neutropenia	27 (56)	17 (35)
Thrombocytopenia	23 (48)	12 (25)
Anemia	23 (48)	11 (23)
Febrile neutropenia	6 (13)	6 (13)
Non-hematologic toxicities		
Constipation	13 (27)	1 (2)
Nasopharyngitis	13 (27)	0 (0)
Hypoalbuminemia	12 (25)	2 (4)
Edema	12 (25)	0 (0)
Pyrexia	11 (23)	1 (2)
Stomatitis	11 (23)	1 (2)
Headache	11 (23)	0 (0)
Insomnia	10 (21)	0 (0)
Alanine aminotransferase increased	10 (21)	1 (2)
Nausea	9 (19)	1 (2)
Decreased appetite	9 (19)	4 (8)
Malaise	8 (17)	0 (0)
Aspartate aminotransferase increased	8 (17)	0 (0)
Rash	8 (17)	0 (0)
Abnormal hepatic function	7 (15)	3 (6)
Herpes zoster	7 (15)	1 (2)
Pruritus	7 (15)	0 (0)
Protein in urine	7 (15)	0 (0)
Vomiting	6 (13)	0 (0)
Cytomegalovirus infection	5 (10)	0 (0)
Pneumonia	5 (10)	4 (8)
Hyponatremia	5 (10)	4 (8)
Decreased weight	5 (10)	1 (2)

Twenty-two patients (46%) experienced serious AEs, most commonly infections (*n* = 8). Pneumonia (*n* = 4) was the only serious infection reported in > 1 patient. Secondary B cell lymphoma was reported in five patients; all were women aged 65 to 75 years, and three entered the study with AITL and two with PTCL-NOS. In four of five patients, lymphoma cells were positive for EBV encoded RNA-1 on in situ hybridization. Based on an exploratory analysis, the patients who developed secondary B cell lymphoma had received forodesine for a median of 11.6 months (range, 2.2–16.6 months); the median duration from forodesine initiation to development of secondary B cell lymphoma was 14.3 months (range, 6.7–16.6 months). Median trough lymphocyte counts in patients who did (*n* = 5) or did not (*n* = 43) develop secondary B cell lymphoma were 87 per mm^3^ (range, 51–120) and 71 per mm^3^ (range, 0–731), respectively. Median trough CD4^+^ lymphocyte counts in patients who did (*n* = 5) or did not (*n* = 42) develop secondary B cell lymphoma were 44 per mm^3^ (range, 18–162) and 51/mm^3^ (range, 5–3274), respectively. Outcome for patients who developed secondary B cell lymphoma are shown in Table 3. One patient achieved CR to treatment for secondary B cell lymphoma and survived with PTCL at the time of the final analysis. Three patients died of lymphoma (PTCL and/or secondary B cell lymphoma), and the outcome of one patient is unknown.

**Table 3:** Outcomes for patients who developed secondary B cell lymphoma

Disease classification	Sex	Age^a^	Duration of forodesine administration (days)	Duration to development of sBCL^b^ (days)	Treatments for sBCL/response	Outcome
AITL	F	71	447	450	R-DeVIC/CR	Alive with AITL
AITL	F	70	171	203	R-CHOP/PD	Died from lymphoma (AITL, sBCL)
AITL	F	76	67	436	PSL/unknown	Died from lymphoma (AITL, sBCL)
PTCL, NOS	F	72	353	281	PSL/unknown	unknown
PTCL, NOS	F	65	505	506	R-COP/PD R-CHOP/PD DEX/PD	Died from lymphoma (PTCL, NOS, sBCL)

*AITL* angioimmunoblastic T cell lymphoma; *COP* rituximab plus cyclophosphamide, vincristine, prednisone; *CR* complete response; *F* female; *PD* progression disease; *PSL* prednisolone; *PTCL* peripheral T cell lymphoma; *R-CHOP* rituximab plus cyclophosphamide, doxorubicin, vincristine, prednisone; *R-DeVIC* rituximab plus dexamethasone, etoposide, ifosfamide, carboplatin; *R-DEX* dexamethasone; *R-GDP* rituximab plus gemcitabine, dexamethasone, cisplatin; *sBCL* secondary B cell lymphoma

^a^Age at the time of informed consent

^b^Duration from initial forodesine administration to development of sBCL

### Efficacy

**Fig. 3:**
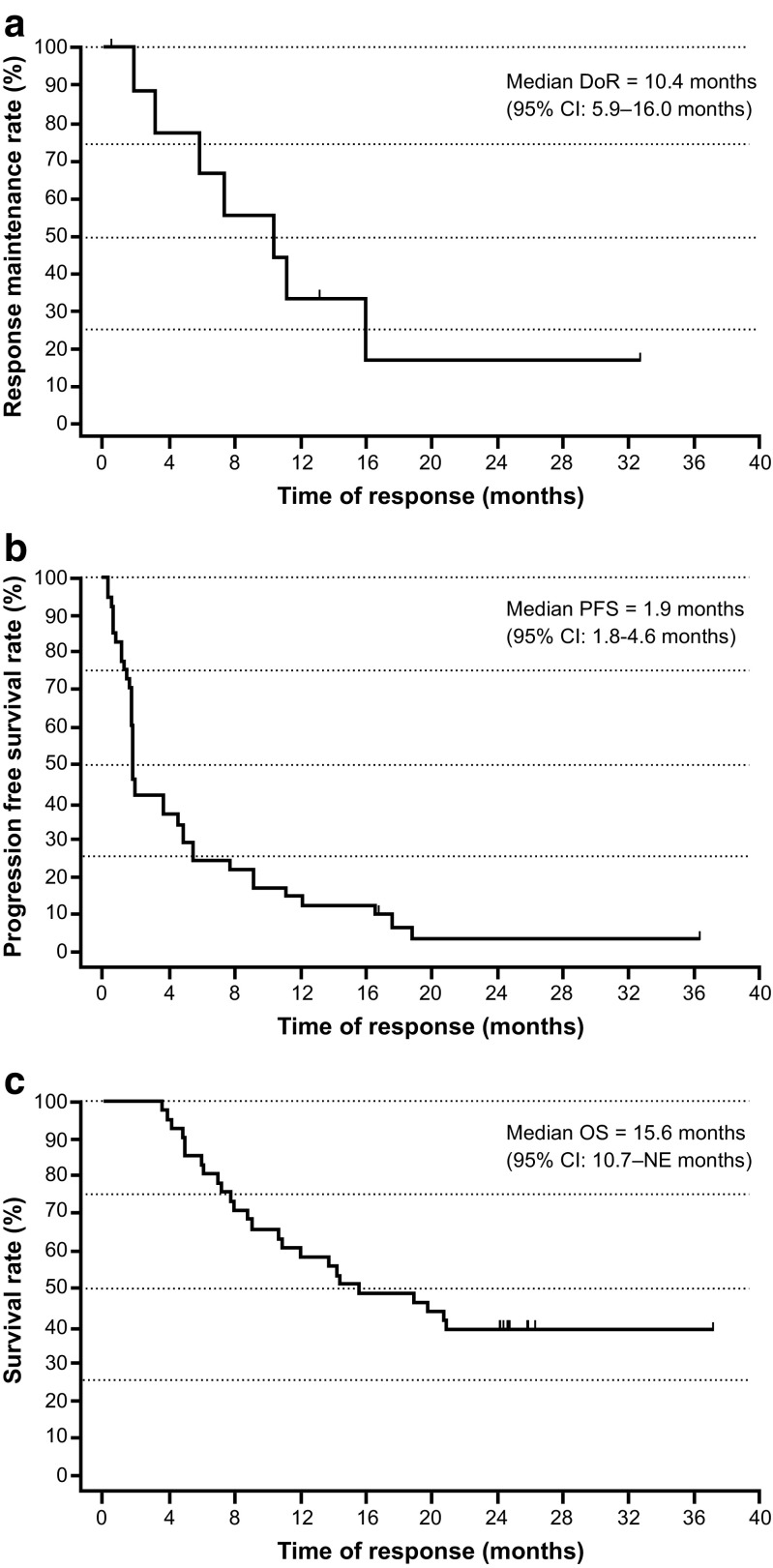
Duration of response (*n* = 10 responding patients) (**a**), progression-free survival (**b**), and overall survival (OS) (**c**) among evaluable phase 2 patients (*n* = 41)

**Fig. 4:**
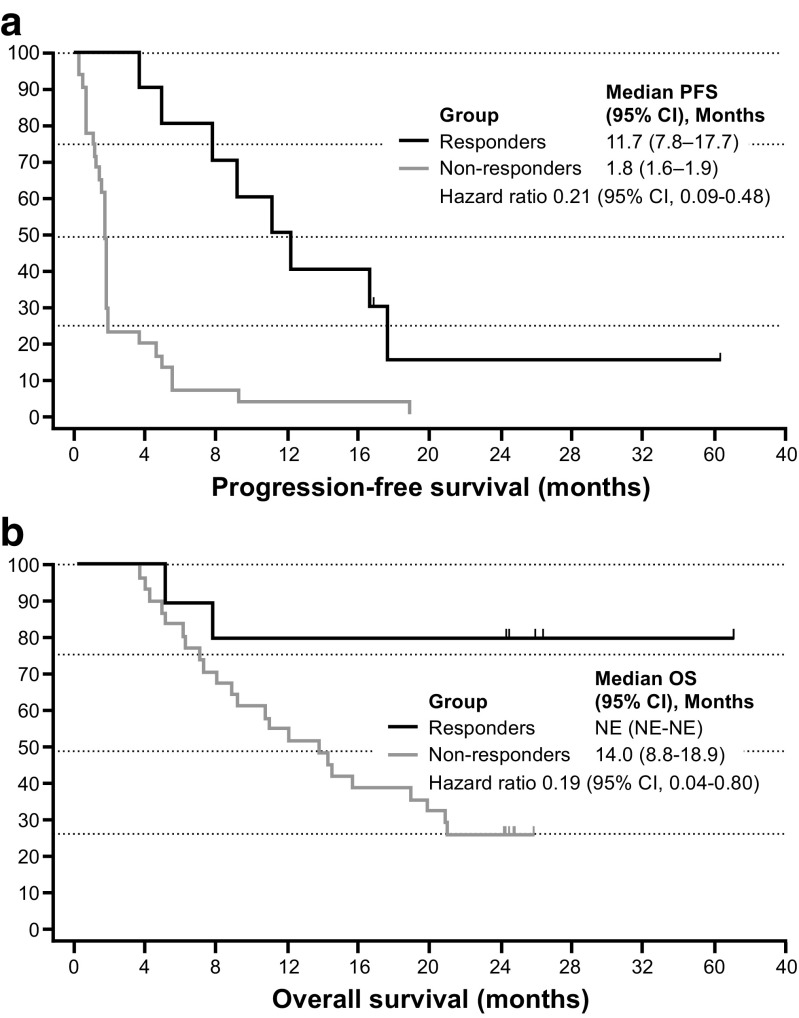
Progression-free survival (**a**) and overall survival (**b**) among responders and non-responders in the phase 2 populations. *CI* confidence interval, *NE* not estimable

Among the 41 evaluable patients in phase 2, the ORR (IEAC assessment) for the primary analysis was 22% (90% CI 12–35%), and included four with CR (10%) and five with PR (12%) (Table 4), which was significantly greater than the predefined 10% threshold rate (*P* = 0.018). The investigator-assessed ORR was 22%, and included three patients with CR and six with PR. One of four patients in phase 1 also achieved a PR. Furthermore, the ORR at the final analysis was 25% (90% CI 14–38%), and included four patients with CR (10%) and six with PR (15%) (Table 4). The median time to response was 2.8 months (range, 1.8–12.8 months), and the median time to treatment failure was 2.2 months (95% CI 1.8–5.0 months) (data not shown). Median DoR was 10.4 months (95% CI 5.9–16.0 months) (Fig. 3a), median PFS was 1.9 months (95% CI 1.8–4.6 months) (Fig. 3b), and median OS was 15.6 months (95% CI 10.7–NE months) (Fig. 3c) among evaluable patients. Two-year OS was 39%. Compared with non-responders, the hazard ratios for PFS and OS among responders were 0.21 (95% CI 0.09–0.48) and 0.19 (95% CI 0.04–0.80), respectively (Fig. 4).

**Table 4:** Objective response rate by IEAC assessment

Response, *n* (%)	Phase 2 (*n* = 41)	Phase 1 + 2 (*n* = 45)
Primary analysis		
DCR (CR + PR+ SD)	16 (39)	17 (38)
ORR (CR + PR) 90% CI	9 (22)^a^ 12–35	10 (22) 13–35
CR	4 (10)	4 (9)
PR	5 (12)	6 (13)
SD	7 (17)	7 (16)
PD/RD	24 (59)	26 (58)
Not evaluable	1 (2)	2 (4)
Final analysis		
DCR (CR + PR+ SD)	16 (39)	17 (38)
ORR (CR + PR) 90% CI	10 (25)^a^ 14–38	11 (24) 14–37
CR	4 (10)	4 (9)
PR	6 (15)	7 (16)
SD	6 (15)	6 (13)
PD/RD	24 (59)	26 (58)
Not evaluable	1 (2)	2 (4)

*CR* complete response, *DCR* disease control rate, *IEAC* Independent Efficacy Assessment Committee, *ORR* objective response rate, *PD* progressive disease, *PR* partial response, *RD* relapsed disease, *SD* stable disease

^a^Uniformly minimum variance unbiased estimator (UMVUE)

**Fig. 5:**
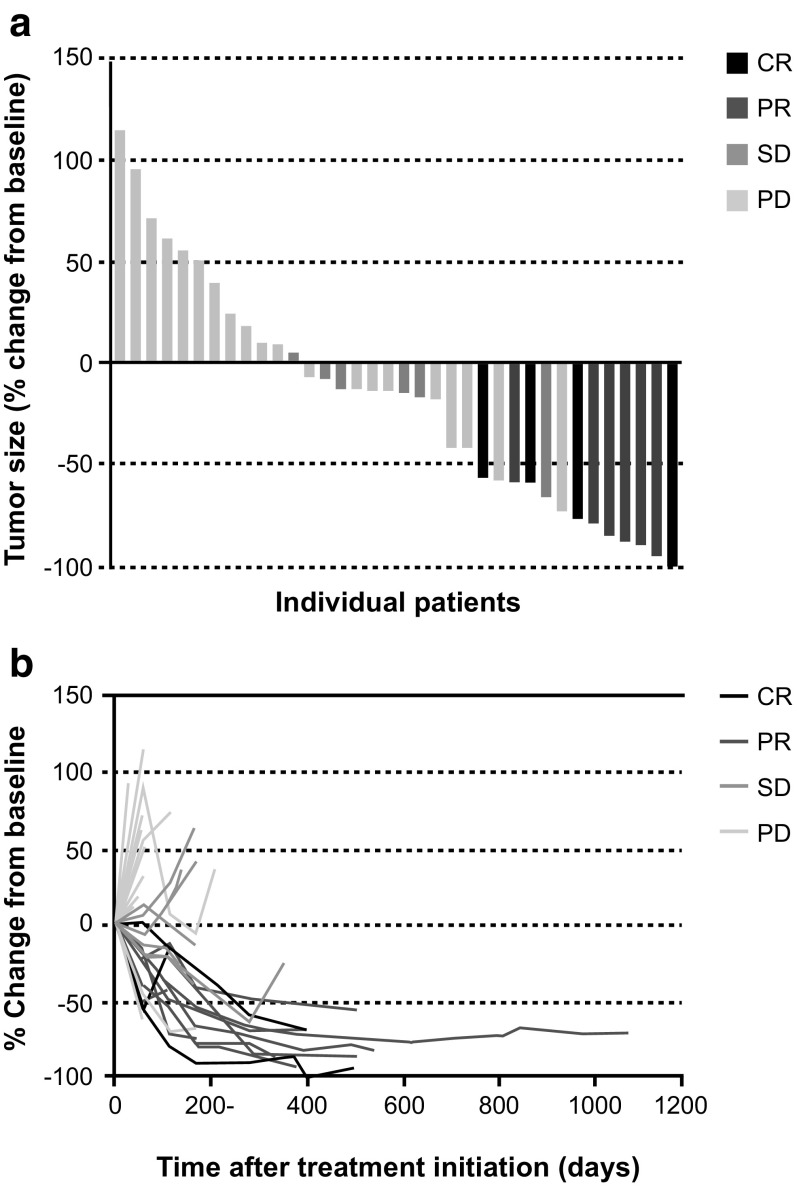
Reduction of target lesions measured by the sum of the products of the greatest diameters in the phase 2 populations: waterfall plot of maximum reduction (**a**) and target lesion reduction rate (**b**). *CR* complete response, *PD* progressive disease, *PR* partial response, *SD* stable disease

For the major PTCL subtypes in the phase 1 and 2 cohorts combined, the ORR was 33% (95% CI 13–59%) among 18 evaluable patients with AITL and 23% (95% CI 8–45%) among 22 evaluable patients with PTCL-NOS. ORRs > 30% were observed in several predefined subgroups, including age < 65 years (6/16; 38%), two prior treatment regimens (3/9; 33%), stage III disease (7/19; 37%), and low/normal lactate dehydrogenase (9/25; 36%) (Table 5). In general, patients with CR and PR showed a progressive reduction in target tumor size over time after starting forodesine (Fig. 5).

**Table 5:** Subgroup analysis of objective response rate, full analysis set

Subgroup	*n*/*N*^a^	ORR (95% CI), %
All evaluable patients in phases 1 + 2	11/45	24 (13–40)
Sex		
Men	8/30	27 (12–46)
Women	3/15	20 (4–48)
Age group		
< 65 years	6/16	38 (15–65)
≥ 65 years	5/29	17 (6–36)
No. of previous anti-tumor regimens		
1	5/19	26 (9–51)
2	3/9	33 (8–70)
3	3/10	30 (7–65)
≥ 4	0/7	0 (0–41)
Histological classification		
Peripheral T cell lymphoma, NOS	5/22	23 (8–45)
Angioimmunoblastic T cell lymphoma	6/18	33 (13–59)
Ann Arbor stage^b^		
Stage I	0/1	0 (0–98)
Stage II	2/11	18 (2–52)
Stage III	7/19	37 (16–62)
Stage IV	2/13	15 (2–45)
ECOG performance status		
0	7/27	26 (11–46)
1	4/18	22 (6–48)
Target lesion SPD		
< 14 cm^2^	8/26	31 (14–52)
≥ 14 cm^2^	3/19	16 (3–40)
LDH		
Low/normal	9/25	36 (18–58)
High	2/20	10 (1–32)

*CI* confidence interval, *ECOG* Eastern Cooperative Oncology Group, *LDH* lactate dehydrogenase, *NOS* not otherwise specified, *ORR* objective response rate, *SPD* sum of the products of the largest diameters of target lesions

^a^Number of patients with objective responses divided by the total number of patients in the category

^b^Except for transformed mycosis fungoides

### Pharmacokinetics

**Fig. 6:**
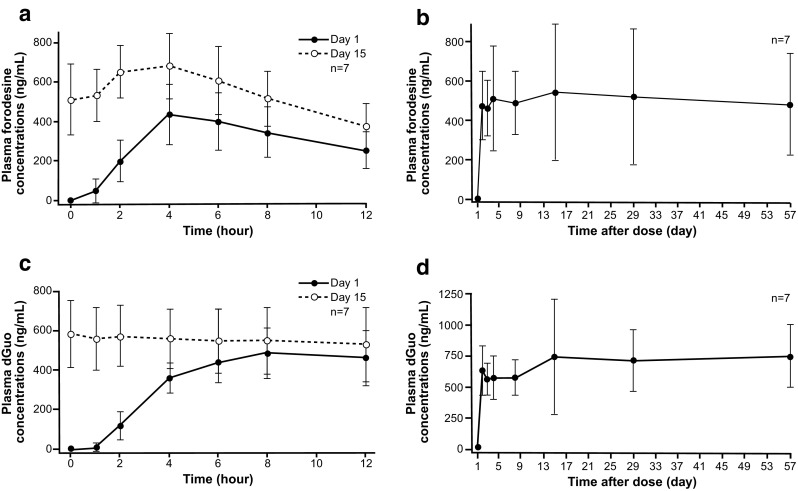
Plasma forodesine and 2′-deoxyguanosine (dGuo) concentrations. Concentration-time profile of plasma forodesine on days 1 and 15 (**a**), trough plasma forodesine concentrations from days 1 to 57 (**b**), concentration-time profile of plasma dGuo on days 1 and 15 (**c**), and trough plasma dGuo concentrations from days 1 to 57 (**d**)

Plasma forodesine concentrations increased over 4 h after the first dose (mean [± standard deviation] *C*_max_, 435.7 [± 152.9] ng/mL) and then decreased gradually (Fig. 6). On day 15, the mean pretreatment concentration was 509.7 ng/mL (± 180.4) and after dosing, again increased over 4 h to a mean of 683.1 ng/mL (± 162.9) before gradually decreasing. Throughout the treatment period, mean trough plasma forodesine concentrations at each time point remained within a range of 460 to 540 ng/mL. The AUC_last_ values after dosing on days 1 and 15 were 3540 and 6520 ng h/mL, indicating an accumulation ratio of 1.8. Plasma dGuo levels increased over 8 h after forodesine dosing, with mean trough concentrations at each time point within a range of 551 to 840 ng/mL. Plasma forodesine and dGuo concentrations on days 1 and 15 showed a positive relationship according to a maximum drug effect model.

## Discussion

This study demonstrated that forodesine has promising single-agent activity and led to its approval in Japan for treatment of relapsed/refractory PTCL. The ORR (22–25%) is comparable with ORRs reported in phase 2 studies of several recently approved agents for PTCL, including pralatrexate, romidepsin, and belinostat [10–12]. Differences in patient populations, histopathologic subtype distributions, disease status, and pretreatment characteristics make comparisons across studies difficult. Our study cohort mostly consisted of patients with PTCL-NOS and AITL; the ORR was numerically higher for those with AITL (33%) than for those with PTCL-NOS (23%). In a recent phase 2 study of patients with relapsed/refractory PTCL, lenalidomide demonstrated an ORR of 22%, and also showed a higher ORR in the AITL subset (31%) [24]. Given etiologic differences underlying the various PTCL histologies, it is plausible that specific agents may be used preferentially for specific PTCL subtypes. Indeed, brentuximab vedotin is highly active in patients with relapsed/refractory CD30^+^ ALCL but has less activity against other PTCL subtypes [15, 16]. Gemcitabine also showed promising activity in a small study of patients with PTCL-NOS and mycosis fungoides [25].

The median time to objective response was 2.8 months, and responses to forodesine appeared durable. In the final data analysis, the ORR was 25% (one patient reached PR after 13 months of administration) and median DoR was 10.4 months (range, 5.9–16.0 months).

The safety profile of forodesine 300 mg twice-daily was acceptable. Although dose delays because of AEs occurred in 35% of patients, dose reduction was only needed in 1 patient (2%), and discontinuation due to AEs occurred in 11 patients (23%). Toxicity consisted mostly of lymphopenia and other hematologic AEs; non-hematologic toxicities were generally mild/moderate in severity. The high rate of lymphopenia is thought to reflect the mechanism of action of forodesine. By inhibiting PNP, forodesine induces lymphocyte apoptosis (mainly T cells), leading to a reduction in lymphocyte counts and causing an immunosuppressive effect that may result in an increased risk of infection and secondary B cell lymphoma.

In this study, five patients developed secondary B cell lymphoma, of whom three had AITL and two had PTCL-NOS, consistent with the general distribution in our study cohort. EBV-driven B cell lymphoproliferation and EBV-related B cell lymphoma secondary to immunosuppression have been reported in patients with AITL and PTCL-NOS [26, 27]. Clonal expansion of EBV-negative B cells has also been described in patients with PTCLs [28, 29]. EBV status was not assessed at enrollment in our study; patients were not treated with anti-viral agents as prophylaxis, and all patients had received prior immunosuppressive chemotherapy. Thus, it cannot be excluded that the secondary lymphomas were already evolving before forodesine initiation. No clear difference was observed between total lymphocyte and CD4^+^ lymphocyte counts for patients who did or did not develop secondary B cell lymphoma, and risk factors for development of secondary B cell lymphoma were not identified in this study. Future studies should investigate whether EBV status at baseline or during forodesine treatment influences risk of secondary B cell lymphoma. In addition, sufficient attention must be placed on risk of opportunistic infection given that forodesine’s mechanism of action leads to T cell reductions.

In conclusion, forodesine has clinically meaningful single-agent activity, with durable responses, and a manageable safety profile in patients with relapsed PTCL. Compared with PTCL options that require intravenous infusion with frequent or prolonged clinic visits, the oral formulation makes forodesine easier to administer and, in turn, may be more convenient and less burdensome to patients. New therapeutic strategies with forodesine, including combination therapy, are being considered.
